# A standard, single dose of inhaled terbutaline attenuates hyperpnea-induced bronchoconstriction and mast cell activation in athletes

**DOI:** 10.1152/japplphysiol.00700.2015

**Published:** 2016-02-04

**Authors:** A. J. Simpson, J. R. Bood, S. D. Anderson, L. M. Romer, B. Dahlén, S.-E. Dahlén, P. Kippelen

**Affiliations:** ^1^Centre for Human Performance, Exercise and Rehabilitation, College of Health and Life Sciences, Brunel University London, United Kingdom;; ^2^Unit for Experimental Asthma Research, Institute of Environmental Medicine, Karolinska Institutet, Stockholm, Sweden;; ^3^Unit for Clinical Asthma Research, Department of Internal Medicine, Karolinska University Hospital Huddinge, Stockholm, Sweden;; ^4^Centre for Allergy Research, Karolinska Institutet, Stockholm, Sweden; and; ^5^Department of Respiratory and Sleep Medicine, Royal Prince Alfred Hospital, Sydney, Australia

**Keywords:** inhaled β_2_-agonist, exercise-induced bronchoconstriction, eucapnic voluntary hyperpnea, prostaglandin D_2_

## Abstract

Release of bronchoactive mediators from mast cells during exercise hyperpnea is a key factor in the pathophysiology of exercise-induced bronchoconstriction (EIB). Our aim was to investigate the effect of a standard, single dose of an inhaled β_2_-adrenoceptor agonist on mast cell activation in response to dry air hyperpnea in athletes with EIB. Twenty-seven athletes with EIB completed a randomized, double-blind, placebo-controlled, crossover study. Terbutaline (0.5 mg) or placebo was inhaled 15 min prior to 8 min of eucapnic voluntary hyperpnea (EVH) with dry air. Pre- and postbronchial challenge, urine samples were analyzed by enzyme immunoassay for 11β-prostaglandin F_2α_ (11β-PGF_2α_). The maximum fall in forced expiratory volume in 1 s of 14 (12–20)% (median and interquartile range) following placebo was attenuated to 7 (5–9)% with the administration of terbutaline (*P* < 0.001). EVH caused a significant increase in 11β-PGF_2α_ from 41 (27–57) ng/mmol creatinine at baseline to 58 (43–72) ng/mmol creatinine at its peak post-EVH following placebo (*P* = 0.002). The rise in 11β-PGF_2α_ was inhibited with administration of terbutaline: 39 (28–44) ng/mmol creatinine at baseline vs. 40 (33–58) ng/mmol creatinine at its peak post-EVH (*P* = 0.118). These data provide novel in vivo evidence of mast cell stabilization following inhalation of a standard dose of terbutaline prior to bronchial provocation with EVH in athletes with EIB.

## Abstract (precis)

This study provides the first in vivo evidence for a mast cell stabilizing effect of the short-acting inhaled β_2_-adrenoceptor agonist terbutaline, when administered prophylactically at a clinically recommended dose (0.5 mg) before bronchial provocation with dry air. Our data therefore support the proposal that β_2_-adrenoceptor agonist-mediated mast cell stabilization is a major contributor to bronchoprotection in individuals with exercise-induced bronchoconstriction.

## NEW & NOTEWORTHY

*This study provides the first in vivo evidence for a mast cell stabilizing effect of the short-acting inhaled β_2_-adrenoceptor agonist terbutaline, when administered prophylactically at a clinically recommended dose (0.5 mg) before bronchial provocation with dry air. Our data therefore support the proposal that β_2_-adrenoceptor agonist-mediated mast cell stabilization is a major contributor to bronchoprotection in individuals with exercise-induced bronchoconstriction*.

exercise-induced bronchoconstriction (EIB) is the transient narrowing of airways that occurs during or shortly after strenuous exercise. The majority of individuals with asthma experience EIB (33), and EIB is also prevalent in children (7, 29) and elite athletes (17). The Global Initiative for Asthma guidelines suggest that inhaled short-acting β_2_-adrenoceptor agonists should be prescribed to all individuals with asthma for use “as needed reliever” and recommends their use for the short-term prevention of EIB (27). A recent Cochrane review of 45 studies revealed that inhaled short-acting β_2_-adrenoceptor agonists, when taken prophylactically, reduce postexercise bronchoconstriction by 66% in individuals with EIB (9). The primary mechanism of this protection is believed to be functional antagonism, whereby β_2_-adrenoceptor-induced relaxation of the bronchial smooth muscle opposes the contractile effects of the various mediators of bronchoconstriction (2).

In 1975 and 1976, Anderson and colleagues (4, 5) reported that β_2_-adrenoceptor agonists given by inhalation in low doses were superior to tablets (given in much higher doses) in preventing EIB, even though both formulations induced bronchodilatation. Those authors therefore proposed that the aerosol had an additional site of action to the smooth muscle and prevented EIB by delivering a concentration of drug sufficient to stabilize mast cells in the airways and inhibit the release of “bronchoconstrictor substances.”

Evidence of mast cell activation associated with exercise and hyperpnea of dry air is now well established by the finding of an increase in urinary excretion of 11β-prostaglandin F_2α_ (11β-PGF_2α_), i.e., a main metabolite of prostaglandin (PG) D_2_, which is produced almost exclusively from mast cells (31, 34, 35, 37, 38, 46). The proposal that inhalation of β_2_-adrenoceptor agonists may prevent EIB by inhibiting release of mast cell mediators is supported by in vitro observations of an inhibition of IgE-dependent release of PGD_2_ and histamine from mast cells with the short-acting β_2_-adrenoceptor agonist salbutamol (19). In vivo, the long-acting β_2_-adrenoceptor agonist salmeterol has been shown to inhibit mast cell mediator release following provocation with increasing doses of lysine-aspirin in patients with aspirin-induced asthma (54). Further, a large dose of the long-acting β_2_-adrenoceptor agonist formoterol has been shown to reduce the release of 11β-PGF_2α_ following bronchial provocation with dry powder of mannitol in patients with asthma (15). Whether a single, standard dose of short-acting β_2_-adrenoceptor agonist—the mainstay treatment for EIB prevention—has a similar stabilizing effect on mast cells during EIB remains to be established.

The aim of this investigation was to test the efficacy of a single, clinically recommended dose of an inhaled short-acting β_2_-adrenoceptor agonist at inhibiting mast cell mediator release following hyperpnea with dry air. Our hypothesis was that premedication with 0.5 mg of terbutaline would attenuate the increase in urinary excretion of 11β-PGF_2α_ following 8 min of eucapnic voluntary hyperpnea (EVH) in athletes with EIB.

## METHODS

### 

#### Subjects.

Part of the current methodology has been published elsewhere to address a separate research question (53). Twenty-seven athletes with EIB completed the study. EIB was determined by a fall of ≥10% in forced expiratory volume in 1 s (FEV_1_) following an 8-min EVH challenge during a screening visit. Participants were nonsmokers, were free from any respiratory infections for 4 wk prior to the study, and had no known chronic medical condition other than asthma and/or EIB. Alcohol, caffeine, and exercise were withheld on the day of testing, and medications were withheld as follows: short-acting β_2_-agonist treatments for a minimum of 8 h, long-acting β_2_-agonist treatments for 24 h, inhaled corticosteroid treatments for 12 h, combination therapies of long-acting β_2_-agonist plus inhaled corticosteroid treatments for 24 h, and nonsteroidal anti-inflammatory medication for 7 days. Participants provided written informed consent after the study protocol, and potential risks were explained. The study was approved by the United Kingdom National Health Service Research Ethics Committee (NHS REC reference number 10/H0716/30).

#### Experimental design.

The study used a randomized, double-blind, placebo-controlled, crossover experimental design with two experimental visits. The experimental visits were completed within 3 wk and separated by at least 2 days. Urine samples were collected at baseline and at 30 and 60 min following EVH with dry air on two separate days: one following treatment with 0.5 mg of terbutaline and one after administration of a placebo. Urine samples were analyzed for 11β-PGF_2α_. The primary end points were the change in the concentration of urinary 11β-PGF_2α_ and the maximum percent fall in FEV_1_ following EVH.

To standardize for fluctuations in lung function throughout the day (42), all experimental visits commenced between 8.00 and 11.00 a.m. Upon arrival, participants performed spirometry according to American Thoracic Society/European Respiratory Society guidelines (40). A single 0.5 mg dose of terbutaline was then administered via a dry powder inhaler (Bricanyl Turbohaler, Astra Zeneca, London, U.K.). An empty demonstration Turbohaler was used for administration of the placebo. The active drug (or placebo) was administered by one deep, hard inhalation through the inhaler, followed by a 10-s breath hold. Spirometry was repeated 10 min posttreatment.

The EVH challenge began 15 min after treatment administration. The test consisted of 8 min of dry air hyperpnea at a target ventilation of 85% predicted maximum voluntary ventilation (MVV; calculated as 30 × baseline FEV_1_) (1), with the ventilation achieved during the first visit used as the target ventilation for the second visit. The test was performed on a commercially available system (Eucapsys, SMTEC, Nyon, Switzerland) that delivered a dry gas mixture containing 5% CO_2_, 20% O_2_, and balance N_2_. Subjects recovered spontaneously from the EVH challenge, and spirometry was repeated in duplicate at 2, 5, 10, 15, 20, 30, and 60 min postchallenge. The highest of two repeatable FEV_1_ values was kept for analysis. The maximum fall in FEV_1_ was expressed as a percentage from the posttreatment value. The degree of bronchoprotection afforded by terbutaline was calculated by subtracting the maximum percent fall in FEV_1_ on the drug treatment day from the maximum percent fall in FEV_1_ on the placebo day, and expressing it as a percentage of the placebo.

The atopic status of the participants was determined by a standard skin prick test (12) conducted 40 min post-EVH during the first experimental visit. The following allergens were tested: cat hair, timothy grass, and house dust mite (ALK-Abello, Reading, U.K.). Histamine and saline were used as positive and negative controls, respectively. A test was deemed positive if the reaction wheal was ≥3 mm.

Ingestion of water was standardized throughout the experimental visits. One hour prior to the visits, subjects were asked to drink 200 ml of water. They were given a further 400 ml upon arrival at the laboratory, and then 200 ml every 30 min. Two baseline urine samples were collected: the first on arrival at the laboratory, which was discarded, and the second 30–60 min later and immediately before administration of the drug, which was used as the baseline. Following the EVH challenge, further urine samples were collected at 30 and 60 min. The urine samples were stored without preservatives at −80°C. The urine samples were analyzed for 11β-PGF_2α_ using commercially available EIA reagents (Cayman Chemical, Ann Arbor, MI) as described elsewhere (10). Urinary data on excretion of 11β-PGF_2α_ were normalized in relation to excretion of creatinine using the modified Jaffe colorimetric method [as done previously (10)] and expressed as nanograms of excreted mediator per micromole of creatinine.

#### Statistics.

Sample size was based on a previous study in which the mast cell stabilizing effect of sodium cromoglycate was investigated during EVH in athletes with EIB (34). With a risk alpha of 5%, a risk beta of 95%, and using the effect size from Kippelen and colleagues (34), analysis of 16 urine samples was required [G*Power3 software (26)].

Data were tested for normality using the Shapiro-Wilk test. Baseline FEV_1_ (l), changes in FEV_1_ following treatment (%), ventilation during EVH (l), level of bronchoprotection (%), and stature of the participants were normally distributed and data are presented as means ± SD. Differences between conditions for these variables were analyzed using paired sample *t*-tests. All other data were nonnormally distributed and therefore presented as median and interquartile range (Q1–Q3). Differences between conditions for maximum change in FEV_1_ (%) and urinary 11β-PGF_2α_ concentrations postchallenge were analyzed using Wilcoxon signed-rank tests. Differences across time were analyzed using Friedman two-way analysis of variance by ranks. In cases of statistical significance, Wilcoxon signed-rank tests were used to identify where differences occurred. The relationship between the change in FEV_1_ (%) and the change in urinary 11β-PGF_2α_ following EVH was initially assessed via Spearman correlation tests, in each experimental condition, separately; this was then followed by a multiple regression analysis for within-subject repeated measures (8). All statistical analyses were conducted using SPSS 20 (Chicago, IL). The level of significance was set at *P* < 0.05.

## RESULTS

### 

#### Participant characteristics.

Age, stature, and body mass of the participants were 23 (19–32) yr, 174 ± 8 cm, and 71 (67–79) kg, respectively. Participants were involved in the following sports: athletics (*n* = 13), cycling (*n* = 1), football (*n* = 4), rugby (*n* = 1), cricket (*n* = 1), netball (*n* = 1), and rowing (*n* = 6). They were training for 8 (5–10) h/wk in aerobic activities, and had 8 (5–12) yr experience in their sport. Medical diagnosis, current treatment, and atopic status are presented in Table 1.

**Table 1.** Participant characteristics

**ID**	**Gender**	**Atopic Status**	**Baseline FEV**_**1**_**, % Predicted**	**Previous Diagnosis**	**Treatment**	**Prescribed ICS Dose, μg/day**
1	M	+	95	Asthma + EIB	Combination, SABA	1,600
2	M	+	92	Asthma	SABA, ICS	200
3	M	+	99	Asthma + EIB	Combination	400
4	F	+	108	EIB	SABA, ICS	200
5	M	+	97	−	−	−
6	F	+	92	Asthma	SABA, ICS	200
7	M	+	93	Asthma + EIB	SABA	−
8	F	+	93	Asthma	Combination	400
9	M	+	99	Asthma	SABA, ICS	600
10	M	−	99	−	−	−
11	M	+	85	Asthma	−	−
12	F	+	103	Asthma	−	−
13	M	+	109	EIB	SABA	−
14	F	−	99	Asthma + EIB	Combination	400
15	F	−	109	−	−	−
16	M	+	94	Asthma	SABA	−
17	F	−	96	Asthma	SABA	−
18	M	+	94	Asthma	SABA	−
19	M	+	111	Asthma	SABA, ICS	200
20	F	+	102	EIB	SABA, ICS	200
21	F	+	86	Asthma	SABA	−
22	F	−	88	EIB	−	−
23	F	+	82	Asthma + EIB	SABA	−
24	M	+	91	Asthma	SABA	−
25*	M	+	102	Asthma	SABA	−
26*	M	−	85	Asthma	Combination	200
27*	M	+	109	Asthma + EIB	ICS	100

Atopic status: +, positive skin prick response to house dust mite, timothy grass, and/or cat hair; FEV_1_ % predicted, baseline forced expiratory volume in 1 s expressed relative to the predicted value (50); EIB, exercise-induced bronchoconstriction; SABA, short-acting β_2_-agonist; LABA, long-acting β_2_-agonist; ICS, inhaled corticosteroids; combination, combination therapy of LABA and ICS;

* participants who had urinary 11β-PGF_2__α_ concentrations below the level of detection.

#### Baseline lung function and ventilation during EVH.

Baseline FEV_1_ was not significantly different between conditions: 3.68 ± 0.65 and 3.65 ± 0.64 liters in the placebo and terbutaline conditions, respectively (*P* = 0.129). Terbutaline had a small, but statistically significant (*P* < 0.001) bronchodilator effect: FEV_1_ increased by 5 ± 3% with administration of terbutaline. No such bronchodilator effect was noticed after the administration of placebo. Ventilation was slightly but significantly (*P* = 0.047) higher with terbutaline: 102 ± 20 vs. 101 ± 20 l/min (78 ± 7 vs. 80 ± 8% of predicted MVV).

#### Airway response to EVH.

**Fig. 1.**
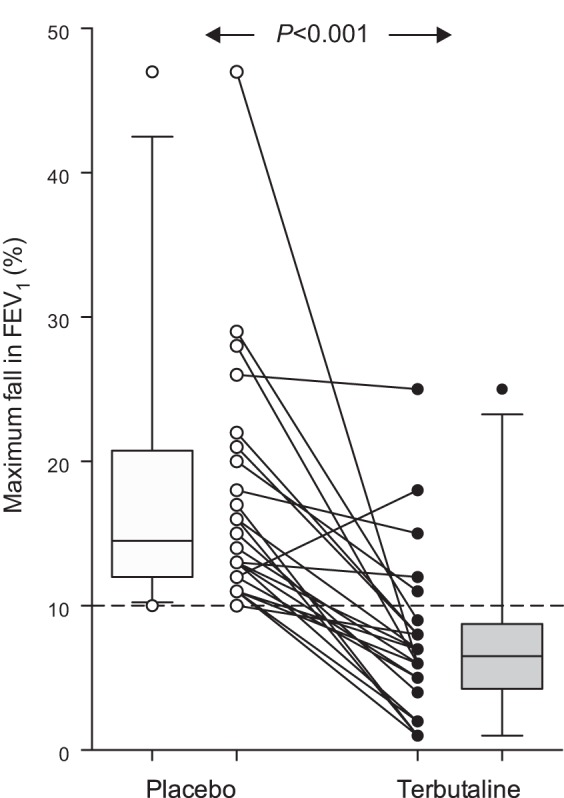
Maximum fall in forced expiratory volume in 1 s (FEV_1_) following 8 min of hyperpnea of dry air in athletes with exercise-induced bronchoconstriction pretreated with 0.5 mg terbutaline (closed symbols) or placebo (open symbols). Individual data are shown; box plots represent group median and interquartile range, with whiskers representing the 5th and 95th percentiles. Values under the broken line (10% fall in FEV_1_) represent complete bronchoprotection (*n* = 22, 81%).

Terbutaline significantly inhibited the airway response to EVH. The maximum fall in FEV_1_ was reduced from 14 (12–20)% in the placebo condition to 7 (5–9)% with the administration of terbutaline (*P* < 0.001). The degree of bronchoprotection afforded by terbutaline was 60 ± 30%, with a range of 0–94%. Terbutaline afforded complete bronchoprotection (<10% fall in FEV_1_ post-EVH) to 22 of the 27 athletes (81%; Fig. 1). The five athletes who did not receive complete bronchoprotection did not differ from the main cohort in regard to their previous medical diagnosis (i.e., asthma with EIB, asthma only, EIB only, or no previous asthma/EIB diagnosis) or their current asthma treatment (i.e., inhaled β_2_-adrenoceptor agonists, inhaled corticosteroids, or no treatment).

#### Urinary 11β-PGF_2α._

Three participants were excluded from statistical analysis because their 11β-PGF_2α_ concentrations were below the level of detection. Of the 24 remaining participants, 18 had complete data sets (i.e., 11β-PGF_2α_ was detectable in all samples), and their data were used to determine the kinetics of urinary 11β-PGF_2α_ excretion post-EVH. An additional six participants had acceptable baseline values and at least one acceptable 11β-PGF_2α_ value post-EVH and were therefore included in the analysis of peak urinary 11β-PGF_2α_ release post-EVH.

#### Kinetics of 11β-PGF_2α_ excretion post-EVH.

**Fig. 2.**
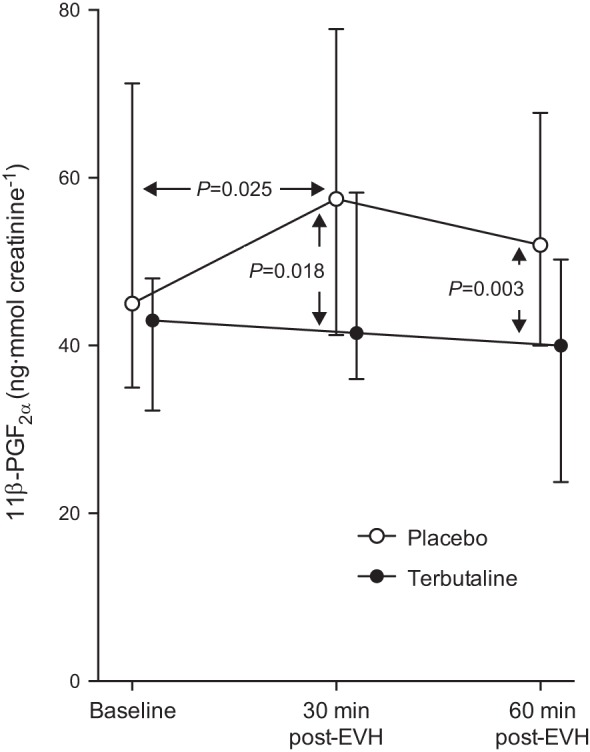
Median ± interquartile range (Q1–Q3) urinary concentration of 11β-prostaglandin F_2α_ (11β-PGF_2α_) at baseline and 30 and 60 min post-eucapnic voluntary hyperpnea of dry air following pretreatment with 0.5 mg terbutaline (closed circles) or placebo (open circles) in 18 athletes with exercise-induced bronchoconstriction.

There was no difference in baseline urinary 11β-PGF_2α_ values between conditions (*n* = 18; *P* = 0.446). A significant time effect was noted in the placebo condition (*P* = 0.002), with an increase in urinary 11β-PGF_2α_ from 45 (35–71) ng/mmol creatinine at baseline to 58 (41–78) ng/mmol creatinine at 30 min postchallenge (*P* = 0.025; Fig. 2). Terbutaline completely inhibited the rise in urinary 11β-PGF_2α_ post-EVH (*P* = 0.446 vs. baseline). Consequently, urinary 11β-PGF_2α_ levels were higher in the placebo vs. the terbutaline condition at 30 min (*P* = 0.018) and 60 min post-EVH (*P* = 0.003; Fig. 2).

#### Peak of urinary 11β-PGF_2α_ post-EVH.

**Fig. 3.**
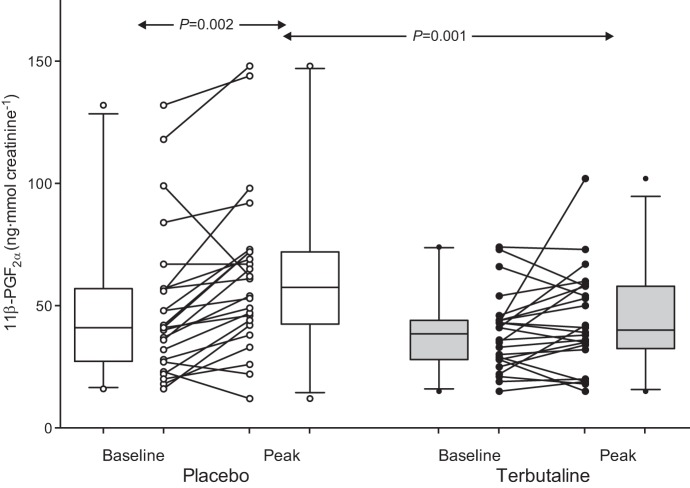
Urinary concentrations of 11β-PGF_2α_ at baseline and after eucapnic voluntary hyperpnea (EVH) of dry air (peak value) following pretreatment with 0.5 mg terbutaline (closed circles) or placebo (open circles) in 24 athletes with exercise-induced bronchoconstriction. Individual data are shown; box plots represent group median and interquartile range, with whiskers representing the 5th and 95th percentiles.

Examination of the baseline and peak 11β-PGF_2α_ values revealed that EVH caused a significant increase in urinary 11β-PGF_2α_ concentration (*n* = 24; *P* = 0.002) that was inhibited with the inhalation of terbutaline (*P* = 0.118, peak vs. baseline; Fig. 3). The inhibition of 11β-PGF_2α_ excretion with terbutaline resulted in a significantly lower peak concentration of 11β-PGF_2α_ in the terbutaline condition compared with the placebo condition (*P* = 0.001; Fig. 3). Similarly, the magnitude of the change in urinary 11β-PGF_2α_ (prechallenge to peak postchallenge) was significantly reduced after pretreatment with terbutaline; from 12 (5–26) ng/mmol creatinine to 3 (−4 to 9) ng/mmol creatinine in the placebo and terbutaline condition, respectively (*P* = 0.033).

Spearman rank correlation coefficient revealed a weak, positive relationship (*r*^2^ = 0.251; *P* = 0.013) between the fall in FEV_1_ and the change in urinary 11β-PGF_2α_ (baseline to peak) post-EVH in the placebo condition. This relationship was absent with the administration of terbutaline (*P* = 0.505). Furthermore, multiple regression analysis revealed no significant relationship between the magnitude of change in urinary 11β-PGF_2α_ and the fall in FEV_1_ post-EVH (*P* = 0.117).

## DISCUSSION

The aim of this study was to determine whether a standard, single dose of inhaled short-acting β_2_-adrenoceptor agonist inhibits mast cell mediator release in a model of EIB. Our results demonstrate that 0.5 mg of terbutaline is able to inhibit the rise in urinary 11β-PGF_2α_ excretion following hyperpnea of dry air in athletes with EIB. These results provide the first in vivo evidence of an inhibition of mast cell activation by a low, clinically recommended dose of inhaled short-acting β_2_-adrenoceptor agonist in response to indirect bronchial provocation.

This study supports previous findings of mast cell activation following hyperpnea of dry air in athletes (34, 35). In vivo evidence using 11β-PGF_2α_ to assess the mast cell stabilizing effect of inhaled β_2_-adrenoceptor agonists in a model of EIB (i.e., bronchial provocation with dry powder mannitol) is limited to the use of a large dose of the long-acting β_2_-adrenoceptor agonist formoterol (15). Long-acting β_2_-adrenoceptor agonists are, however, not recommended as monotherapy for the treatment of asthma (27) or EIB (48). We therefore add novel, clinically relevant data, demonstrating that mast cell stabilization can occur following inhalation of a standard, single dose of the short-acting β_2_-adrenoceptor agonist terbutaline.

Mast cell activation in this study was assessed by a change in the urinary concentration of 11β-PGF_2α_. PGD_2_, the parent molecule of 11β-PGF_2α_, is a major cyclooxygenase metabolite of arachidonic acid. In humans, PGD_2_ is produced almost exclusively from mast cells (47), rendering the urinary metabolite 11β-PGF_2α_ a reliable and convenient marker of mast cell activation (47). A rise in urinary excretion of 11β-PGF_2α_ has repeatedly been observed following induced bronchoconstriction in humans, irrespective of the trigger used: allergen (the gold-standard of mast cell dependent bronchoconstriction) (45), exercise (31, 39, 43, 46), EVH (34, 35), or mannitol (14, 15, 37). Further, in vitro work suggests that human mast cells are activated not only by antigen stimulation but also through osmotic stress (28). In the present study, the average ventilation of ∼100 l/min would have caused water loss from the smaller airways and local hyperosmolarity of the airway surface liquid (22). This is relevant because mast cells are more densely located in the peripheral airways (6, 18). Furthermore, an increase in mast cell density has been noted in individuals with asthma and EIB (36), as well as in competitive swimmers (11). An increase in mast cell density in the peripheral airways, combined with the capability of hyperpnea of dry air to create a hyperosmotic environment, may explain the rise in 11β-PGF_2α_ in the placebo condition in the present study.

Importantly, the deposition of terbutaline from a dry powder inhaler also extends to the small airways (44). This may have facilitated the binding of terbutaline with the β_2_-adrenoceptors on the mast cells infiltrated in the peripheral airways of our study participants. In vitro, mast cells treated with the nonselective β-adrenergic agonist isoprenaline displayed increased levels of cAMP (49). Furthermore, this increase in cAMP correlated with a reduction in the release of the mast cell mediators PGD_2_, histamine, and leukotriene C_4_ (49). In vivo, inhaled terbutaline has also been shown to increase plasma cAMP (16). Because pharmacological agents that induce and sustain elevations of intracellular cAMP are well known to supress mast cell secretions (56), it is likely that the inhibitory effect of mast cell mediator release provided by terbutaline was partly mediated by β_2_-adrenoceptor-induced increase of intracellular cAMP.

Our data support the early proposal by Anderson and colleagues (4, 5) that inhaled β_2_-adrenoceptor agonist may attenuate EIB at least partly by inhibiting mast cell activation. We previously showed that inhaled sodium cromoglycate attenuates bronchoconstriction and urinary 11β-PGF_2α_ excretion following EVH (34). Because sodium cromoglycate has no known bronchorelaxant effect on airway smooth muscle (20, 55), we concluded that sodium cromoglycate attenuates hyperpnea-induced bronchoconstriction via mast cell stabilization (34). Similarly here, we propose that the prophylactic effect of terbutaline was partly mediated by inhibition of mast cell mediator release. This new observation in EVH is in keeping with previous data for allergen-induced bronchoconstriction, where albuterol offered significant bronchoprotection in association with the inhibition of mast cell mediator release (32). However, we cannot dismiss other possible modes of action of β_2_-adrenoceptor agonists within the airways. This would be in keeping with the absence of a relationship between bronchoconstriction (i.e., maximum fall in FEV_1_ post-EVH) and urinary 11β-PGF_2α_ excretion (as observed in our multiple regression analysis), which may be explained by interindividual variation in the contribution of alternative modes of action of terbutaline.

Terbutaline has previously been shown to reduce airway plasma exudation (25). Since vascular engorgement and edema in response to hyperpnea are thought to exaggerate airway obstruction in EIB (3), the antiexudative action of β_2_-adrenoceptor agonists may have contributed to bronchoprotection in our study. Furthermore, there is a possibility that the β_2_-adrenoceptor agonist may have limited excessive and viscous mucosal accumulation by increasing cilia beat frequency (23) and improving clearance of alveolar (51) and/or mucociliary fluids (21, 41). Finally, the β_2_-adrenoceptor agonist may have preserved/improved airway caliber due to its well-known effect on airway smooth muscle tone. However, it has previously been reported that large doses of orally administered β_2_-adrenoceptor agonists fail to prevent EIB while providing significant bronchodilatation (4, 5). Given these observations and our results showing a marked reduction in PGD_2_ release, we consider that mast cell stabilization was an important contributor to the bronchoprotection in our experimental set-up.

Mast cell stabilizing treatments are deemed beneficial for the treatment of asthma/airway hyperresponsiveness (AHR) (13). Further, athletes have an increased prevalence of EIB (17), and mast cell activation has been observed in this population during dry air challenge independently to the occurrence of EIB (34, 35). Our findings are therefore of clinical relevance to individuals with asthma/AHR and to athletes. Given that PGD_2_ may perpetuate inflammation via initiation of the migration of eosinophils to the airways (24), the prevention of mast cell mediator release with β_2_-adrenoceptor agonists may interrupt the chronic inflammatory cycle and improve the management of asthma and/or EIB. Importantly, however, a side effect of regular β_2_-adrenoceptor agonist usage may include the development of tolerance to the medication (30). Therefore, changes to the current guidelines for the management of EIB are not recommended at this time; the use of controller agents, such as inhaled corticosteroids, should be promoted when inhaled β_2_-adrenoceptor agonists have to be used frequently (48). It remains to be determined whether tolerance to β_2_-adrenoceptor agonists is associated in vivo with tolerance to their effects at the level of release of mast cell mediators (52).

To conclude, we have shown that the prophylactic administration of 0.5 mg of inhaled terbutaline not only offers a significant degree of bronchoprotection to athletes with EIB, but is also able to inhibit the release of the mast cell mediator PGD_2_ following hyperpnea of dry air. These findings are in keeping with the proposal that the superior efficacy of aerosol formulation of β_2_-adrenoceptor agonist compared with tablets for preventing EIB is the inhibition of release of mast cell mediators (4, 5).

## GRANTS

This work was performed with financial support from the Swedish Medical Research Council, the Heart Lung Foundation, the Vårdal Foundation, the Stockholm County Council Research Funds (ALF), the Swedish Strategic Research Foundation, the KI-SciLifeLab collaborations on Translational Medicine (ChAMP), the Karolinska Institutet, and the World Anti-Doping Agency.

## DISCLOSURES

No conflicts of interest, financial or otherwise, are declared by the author(s).

## AUTHOR CONTRIBUTIONS

Author contributions: A.J.S., J.R.B., and P.K. performed experiments; A.J.S. and J.R.B. analyzed data; A.J.S., J.R.B., S.D.A., S.-E.D., and P.K. interpreted results of experiments; A.J.S. prepared figures; A.J.S. drafted manuscript; A.J.S., S.D.A., L.M.R., B.D., S.-E.D., and P.K. edited and revised manuscript; A.J.S., J.R.B., S.D.A., L.M.R., B.D., S.-E.D., and P.K. approved final version of manuscript; S.D.A., L.M.R., B.D., S.-E.D., and P.K. conception and design of research.
